# Membrane topology analysis of HIV-1 envelope glycoprotein gp41

**DOI:** 10.1186/1742-4690-7-100

**Published:** 2010-11-30

**Authors:** Shujun Liu, Naoyuki Kondo, Yufei Long, Dan Xiao, Aikichi Iwamoto, Zene Matsuda

**Affiliations:** 1 China-Japan Joint Laboratory of Structural Virology and Immunology, Institute of Biophysics, Chinese Academy of Sciences, 15 Datun Road, Chaoyang District, Beijing 100101, P. R. China; 2 Research Center for Asian Infectious Diseases, and 3Division of Infectious Diseases, Advanced Clinical Research Center, Institute of Medical Science, University of Tokyo, 4-6-1, Shirokanedai Minato-ku, Tokyo 108-8639, Japan; 3 Current Address: Department of Pediatrics, Emory University School of Medicine, 2015 uppergate Dr. Atlanta, GA 30322, USA

## Abstract

**Background:**

The gp41 subunit of the HIV-1 envelope glycoprotein (Env) has been widely regarded as a type I transmembrane protein with a single membrane-spanning domain (MSD). An alternative topology model suggested multiple MSDs. The major discrepancy between the two models is that the cytoplasmic Kennedy sequence in the single MSD model is assigned as the extracellular loop accessible to neutralizing antibodies in the other model. We examined the membrane topology of the gp41 subunit in both prokaryotic and mammalian systems. We attached topological markers to the C-termini of serially truncated gp41. In the prokaryotic system, we utilized a green fluorescent protein (GFP) that is only active in the cytoplasm. The tag protein (HaloTag) and a membrane-impermeable ligand specific to HaloTag was used in the mammalian system.

**Results:**

In the absence of membrane fusion, both the prokaryotic and mammalian systems (293FT cells) supported the single MSD model. In the presence of membrane fusion in mammalian cells (293CD4 cells), the data obtained seem to support the multiple MSD model. However, the region predicted to be a potential MSD is the highly hydrophilic Kennedy sequence and is least likely to become a MSD based on several algorithms. Further analysis revealed the induction of membrane permeability during membrane fusion, allowing the membrane-impermeable ligand and antibodies to cross the membrane. Therefore, we cannot completely rule out the possible artifacts. Addition of membrane fusion inhibitors or alterations of the MSD sequence decreased the induction of membrane permeability.

**Conclusions:**

It is likely that a single MSD model for HIV-1 gp41 holds true even in the presence of membrane fusion. The degree of the augmentation of membrane permeability we observed was dependent on the membrane fusion and sequence of the MSD.

## Background

The envelope glycoprotein (Env) of human immunodeficiency virus type-1 (HIV-1) plays a critical role in the early stage of HIV-1 infection. Env is synthesized as a precursor protein, gp160 [1,2], and processed into gp120 and gp41 during transport from the endoplasmic reticulum to Golgi network [3,4]. The gp120 subunit determines host range through its recognition of the receptor and co-receptor complex. The transmembrane protein gp41 mediates the membrane fusion between the host and viral membranes. It is composed of an ectodomain (extracellular domain), a cytoplasmic domain, and a transmembrane domain. The ectodomain has coiled-coil-forming heptad repeats essential for membrane fusion. The cytoplasmic domain contains three amphipathic helices called the lentiviral lytic peptide (LLP) 1, 2 and 3. The LLP-1 and LLP-2 portions have a high hydrophobic moment common to membrane-lytic peptides [5-9].

**Figure 1:**
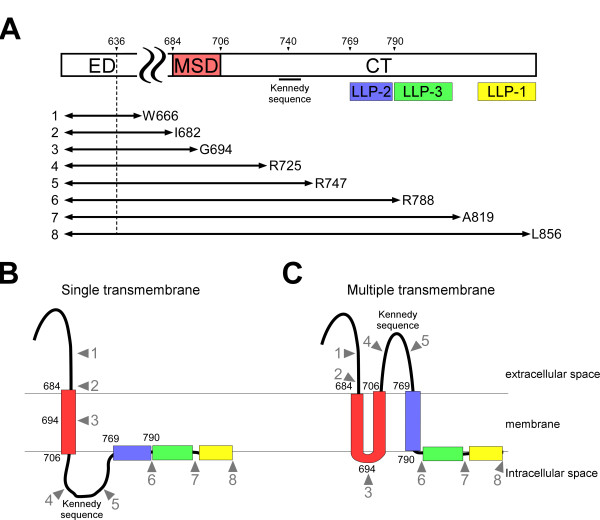
**Schematic representation of Env mutants used in this study and the proposed topology models**. (A) The points of truncation of gp41 were indicated together with a schematic diagram of the gp41 subunit. ED: ectodomain, MSD: membrane-spanning domain, CT: cytoplasmic tail, LLP: lentiviral lytic peptide. The numbering of the amino acid is based on that of the HXB2 strain. The vertical dashed line shows the position of the N-terminus of the gp41 used for the analysis in the bacterial system. The numbers and letters on the right indicate the position and the amino acid residue of the C-terminus. (B and C) Proposed topology models. The grey numbered arrowheads indicate the truncation points of gp41, the numbers and colors correspond to (A).

The transmembrane domain of gp41 was first deduced from the hydropathy plot of Env as a hydrophobic domain [10]. This transmembrane domain, herein referred to as the membrane-spanning domain (MSD), is composed of 23 highly conserved amino acid residues corresponding to amino acid residues 684 to 706 in the HXB2 strain (Figure 1A, B). An in vitro translation study in the presence of microsomal membranes suggested that HIV-1 Env has one MSD [11], as predicted by the hydropathy plot. In that study, the C-terminus of gp41 was assigned to the cytoplasmic side of the cellular membrane [11], hence the gp41 subunit is regarded as a type I membrane protein with a single MSD. Other studies provided data consistent with this single MSD model. For example, two cysteine residues for palmitoylation are located in the cytoplasmic domain: one in the middle of LLP-1 (Cys-838) and the other at the upstream of LLP-2 (Cys-765) [12]. The internalization motif, YXXL (Tyr-769 to Leu-772), at the beginning of LLP-2 [13] also maps to the cytoplasmic domain of the single MSD model.

On the other hand, the mapping of the epitopes for neutralizing antibodies called into question the single MSD model. Some of the epitopes were mapped to the cytoplasmic region which contained the amino acid sequence known as the Kennedy sequence (^724^PRGPDRPEGIEEEGGERDRDRS^745^)[14-16] (Figure 1A). Furthermore, a report using an antibody raised against the LLP-2 portion revealed target binding during membrane fusion when added extracellularly [17]. As antibodies in general are not expected to cross intact membranes, an alternative membrane topology model of gp41 has been suggested in order to assign the mapped epitopes in the extracellular region [16] (Figure 1C). In this alternative model multiple MSDs were proposed because the C-terminus was assumed to be in the cytoplasm. Furthermore, the transmembrane portion of the single MSD model is expected to cross the membrane twice and one of LLPs, LLP2, is a putative third MSD (Figure 1C).

Several studies of the transmembrane portion of the single MSD model showed that it plays a critical role in the modulation of the membrane fusion process, which is an essential step of the HIV-1 life cycle [18-24]. Therefore analysis of the topology and structures of the transmembrane domain of gp41 is critical for our understanding of the mechanism of the membrane fusion. Furthermore the location of the neutralizing epitopes for antibodies is vital for a vaccine development.

In this study we reexamined gp41 topology in two different biological systems; prokaryotic and mammalian systems. The results of prokaryotic and mammalian systems without membrane fusion supported the single MSD model. The results obtained in the mammalian system in the presence of membrane fusion seem to support a transient alteration of the membrane topology of gp41. It is important, however, to note that the effect of the induction of membrane permeability during HIV-1 Env-mediated membrane fusion cannot be excluded. The induction of membrane permeability was reduced by replacing the HIV-1 MSD with that of a foreign protein, CD22.

## Methods

### Plasmid construction

All PCR amplicons were first cloned into pCR4Blunt-TOPO using the TOPO cloning kit (Invitrogen, Carlsbad, CA) and sequences were verified.

For the topology analysis in the prokaryotic system, the expression vector pKMal-p2e was generated. pKMal-p2e has a kanamycin resistance gene derived from pK18 instead of β-lactamase in the context of pMal-p2e (NEB, Beverly, MA). The oligonucleotide adaptor generated by annealing the following two oligonucleotides: 5'-GTACCG AACAAT TACAC AAGCTTC GGATC CTCTAGA GTCGAC CTGCAG GC G-3' and 5'-AGCTC GC CTGCAG GTCGAC TCTAGA GGATCC GAAGCT TGTGTA ATTGTT CG -3' were inserted into pKMal-p2e to modify the multiple cloning site. This modified vector was named as mpKMal-p2e. The green fluorescent protein (GFP) gene as the reporter for the membrane topology was prepared by PCR using GFPopt_1-11 _in pCR4Blunt-TOPO [25] as the template with 5'-GAC **TCTAGA **ATGGTG AGCAAG GGCGAG GAGC-3' and 5'-GCA**CTG CAG**TCA GGTGAT GCCGGC GGCGT-3' as the forward and reverse primer, respectively, and cloned into mpKMal-p2e vector using *Xba*I and *Pst*I sites. The generated vector was named as mpKMalp2e-GFP (Table 1). This plasmid was used for the negative control for the experiment.

**Table 1:** Plasmids used in this study

*Plasmids*	*Description*
For prokaryotic system	

mpKMalp2e-GFP	Multiple cloning site-modified pMalp2e containing *Kan^R ^*and Green fluorescent protein genes

mpKMalp2e-gp41-1-GFP	mpKMalp2e-GFP with C-terminally truncated gp41 at W666

mpKMalp2e-gp41-2-GFP	mpKMalp2e-GFP with C-terminally truncated gp41 at I682

mpKMalp2e-gp41-3-GFP	mpKMalp2e-GFP with C-terminally truncated gp41 at G694

mpKMalp2e-gp41-4-GFP	mpKMalp2e-GFP with C-terminally truncated gp41 at R725

mpKMalp2e-gp41-5-GFP	mpKMalp2e-GFP with C-terminally truncated gp41 at R747

mpKMalp2e-gp41-6-GFP	mpKMalp2e-GFP with C-terminally truncated gp41 at R788

mpKMalp2e-gp41-7-GFP	mpKMalp2e-GFP with C-terminally truncated gp41 at A819

mpKMalp2e-gp41-8-GFP	mpKMalp2e-GFP with full-length gp41

optGFP_1-11_/pET-47md	Modified pET-47b with modified super folder GFP

For mammalian system	

pHIVenv-Halo	The CMV promoter driven mammalian expression vector containing HaloTag gene

pHIVenv-gp41-4-Halo	pHIVenv-Halo containing Env with C-terminally truncated gp41 at R725

pHIVenv-gp41-5-Halo	pHIVenv-Halo containing Env with C-terminally truncated gp41 at R747

pHIVenv-gp41-6-Halo	pHIVenv-Halo containing Env with C-terminally truncated gp41 at R788

pHIVenv-gp41-7-Halo	pHIVenv-Halo containing Env with C-terminally truncated gp41 at A819

pHIVenv-gp41-8-Halo	pHIVenv-Halo containing full-length Env

pHIVenv-gp41-5	Halo-deleted pHIVenv-gp41-5-Halo

pHIVenv-gp41-8	Halo-deleted pHIVenv-gp41-8-Halo

pHIVenv-CD22-gp41-5	The gp41 MSD replaced pHIVenv-gp41-5 with the MSD of CD22

pHIVenv-CD22-gp41-8	The gp41 MSD replaced pHIVenv-gp41-8 with the MSD of CD22

pHook-Halo-GPI	The expression vector of the GPI anchored-HaloTag

pKcTac-Halo	The expression vector of Tac antigen of IL-2 receptor fused with C-terminal HaloTag

pKcTac-FLAG	pKcTacHalo vector whose HaloTag was replaced with 3xFLAG

**Figure 2:**
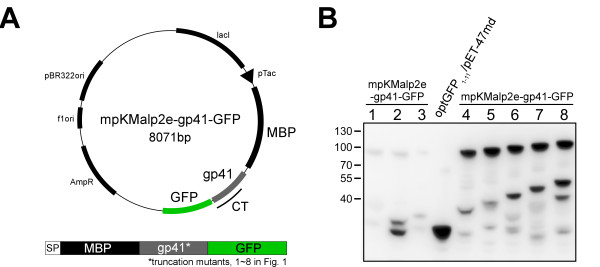
**Constructs used to express recombinant gp41 in the *E. coli *system**. (A) The expression vectors used in this study. The *env *gene is derived from HIV-1 HXB2 strain. gp41 starts amino acid 636 and ends with the various C-terminal positions as indicated in Fig. 1A. The schema below the plasmid map shows the components of the recombinant proteins. The truncated gp41 is preceded by maltose binding protein (MBP) and followed by the topological reporter, green fluorescent protein (GFP). The nomenclatures are as follows: lacI, lacI repressor gene; pTac, a hybrid promoter between trp and lac promoters; CT, cytoplasmic tail; AmpR, ampicilin resistant gene; f1ori, replication origin of f1; pER322ori, replication origin of pBR322 plasmid. (B) Expression of the recombinant proteins. The immunoblotting of bacterial lysates probed with the anti-GFP antibody is shown. The name on the top of each lane indicates the expression vector used (see Table 1).

The near full-length gp41 gene derived from the HIV-1 HXB2 strain was amplified by PCR using pGEM7zf(+)-NB [23] as a template with 535fACC651 (Met):5'-AGT**GGT ACC**GAT GACGCT GACGGT ACAGGC CAGA-3' and 856 rXbaI: 5'-GTC**TCT AGA**TAG CAAAAT CCTTTC CAAGCC CTG-3' as the forward and the reverse primer, respectively. The plasmid that harbors near full-length gp41 in pCR4blunt-TOPO was named as pEnv-HXb2gp41. For the construction of the gp41 mutants, the C-termini were serially truncated, (see Table 1 and Figure 1A), the various gp41 fragments were amplified by PCR using pEnv-HXb2gp41 as a template, with the oligonucleotide 535fACC651 as a forward primer, and the corresponding reverse primer designed for each truncation site. These truncated gp41 fragments were cloned into the vector mpKMalp2e-GFP with *Hind*III, which is present in the gp41 gene, and *Xba*I at the 5' and 3' terminus, respectively of the fragments. Figure 2A shows the resulting mpKMalp2e-gp41-GFP fusion constructs. The plasmid, optGFP_1-11_/pET-47md [26] that expresses GFP in the cytoplasm was used as a positive control.

**Figure 3:**
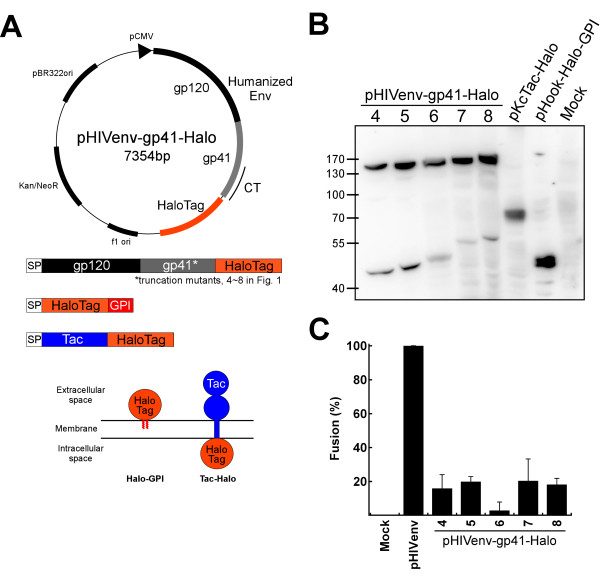
**Constructs used for the expression of reporter proteins in the mammalian system**. (A) The expression vector used in this study. The *env *gene of HXB2 origin was codon-optimized for human genes. The nomenclatures are as follows: pCMV, cytomegalovirus promoter; CT, cytoplasmic tail; HaloTag, Halo7 gene; f1ori, replication origin of f1; Kan/NeoR, kanamycin or neomycin resistant gene; pER322ori, replication origin of pBR322 plasmid. The composition of the fusion protein used in the study was indicated below the plasmid map. The gp41 proteins with different C-terminal truncation points were fused to HaloTag at their C-terminus. The Halo-GPI, and Tac-Halo constructs and their expected membrane topology are shown schematically. (B) The result of immunoblotting with anti-HaloTag antibody. The names of the mammalian expression vector used are indicated above each lane. (C) Analysis of membrane fusion efficiency. The fusion activity of Halo-fused Env was evaluated by the syncytia-forming activity in 293CD4 cells. The percentage of the number of the nuclei included in syncytia was calculated by counting 300 nuclei in total. The constructs tested are indicated at the bottom of each bar; the number indicated the truncation points shown in Fig.1A.

The Halo7 gene was amplified by PCR using pFC14k-HaloTag7 (Promega, Madison, WI) as a template, with 5'- **GTCGAC **GGCGGT GGCGGT AGCGGA TCCGAA ATCGGT ACTG-3' and 5'- **GGTACC **TTAACC GGAAAT CTCCAG AG -3' oligonucleotides as the forward and the reverse primer, respectively. The forward primer contained a *Sal*I site and short linker sequence, Gly_4_Ser, between the *Sal*I site and Halo7 coding region. The reverse primer included an *Acc65*I site. The amplicon was inserted into the pHIVenvOPT vector, containing an envelope gene based on HXB2 strain that was optimized for human codon usage. The vector generated was named as pHIVenv-Halo (Figure 3A). To construct the truncation gp41 mutants for the mammalian analyses, five different positions were chosen as the C-terminal truncation points (Figure 1A and Table 1). The fragments of truncated *env *from *Xmn*I to each termination codon were amplified by PCR using pHIVenvOPT as a template with 5'-**GCTAGC **AAATTA AGAGAAC-3' including the *Sal*I site as the forward primer and the corresponding oligonucleotides at the truncated sites as the reverse primers, respectively. The *env *fragments were inserted into pHIVenv-Halo (Table 1). For the construction of pHIVenv-gp41-5 and pHIVenv-gp41-8, stop codon-containing oligonucleotides generated by annealing 5'-TCGACTGATGAG -3' with 5'-GTACCTCATCAG-3' was replaced with HaloTag gene to delete HaloTag. The Env expression vector with the MSD of CD22 [27] was constructed using PCR and replacement of the original MSD with the MSD of CD22. As for the control plasmids, two other expression vectors were constructed. The glycosylphosphatidylinositol (GPI)-anchored HaloTag gene was constructed as a marker for surface expression of HaloTag (Halo-GPI in Figure 3A and Table 1). The GPI signal is derived from decay accelerating factor of human origin [28]. A Tac antigen, which is alpha subunit of Interleukin-2 receptor and is a single transmembrane protein [29], was fused with HaloTag gene at the C-terminus (Tac-Halo in Figure 3A and Table 1). This construct was used for the expression of the HaloTag protein in the cytoplasm. A derivative of this expression vector for Tac with a FLAG epitope at its C-terminus (Tac-FLAG) was generated by replacing the HaloTag sequence with that for 3xFLAG tag.

### Expression of GFP-fused gp41 proteins and measurement of GFP fluorescence intensity

*E. coli *strain BL21 transformed with mpKMal-p2e carrying serially truncated gp41 genes fused to GFP reporter was grown overnight at 22°C in TAG medium (10 g/L Tryptone, 5 g/L NaCl, 5 g/L Glucose, 7 g/L K_2_HPO_4_, 3 g/L KH_2_PO_4_, 1 g/L (NH_4_)_2_SO_4_, 0.47 g/L Sodium Citrate) with 50 μg/ml kanamycin. The overnight bacterial culture was diluted 1:50 in 4 ml TAG fresh medium containing 50 μg/ml kanamycin and growth was continued at 22°C until the OD_600 _reached 0.2. Cells were grown for overnight in the presence of 0.1 mM IPTG. Subsequently, one ml aliquot of culture was collected and resuspended in 0.5 ml of PBS buffer and the GFP fluorescence intensity was measured by flow cytometry using a FACS Calibur (BD Biosciences, Mississauga, ON). At the same time, another 1 ml aliquot of culture was dispensed for SDS-PAGE and immunoblotting analysis.

### Mammalian cell culture, transfection, labeling, and imaging

The 293FT cells (Invitrogen, Carlsbad, USA) or 293CD4 cells (293 cells constitutively expressing human CD4) [23] were grown in 96-well Matriplates (GE Healthcare, Piscataway, NJ) with Dulbecco's modified Eagle medium (DMEM; Sigma, St. Louis, USA) supplemented with 10% FBS (Hyclone Labs., Logan, UT). In the case of 293FT, 5 μg/ml Geneticine (GiBco, Grand Island, USA) was further supplied. Cells were grown at 37°C in 5% CO_2 _incubator.

DNA transfection of mammalian cells was performed using Fugene HD (Roche, Indianapolis, USA; Fugene HD (μl): DNA(μg): DMEM(μl) = 5:2:200). The transfection mix was incubated for 15 mins at room temperature prior to addition to the cell culture in a drop-wise manner (10 μl per well). After certain hours of transfection the transfected cells were subjected for further analyses as described below.

At the indicated time after transfection, the transfected cells were probed with HaloTag ligands. The starting time point of labeling after transfection was different for different experiments involving a different set of cells and vectors (see the Results section). The labeling was performed as suggested by the manufacturer (Promega). Briefly, the transfected live cells were labeled for 15 mins at 37°C with 1 μM of HaloTag ligand Alexa Fluor 488 (AF488), a membrane-impermeable ligand, or Oregon Green (OG), a membrane permeable ligand, respectively. After labeling, the cells were rinsed three times with 200 μl prewarmed DMEM plus 10% FBS and subsequently incubated at 37°C with 5% CO_2 _for 30 mins. The medium was changed with fresh warm DMEM plus 10% FBS, then images were captured using a confocal microscope (Olympus FluoView FV1000, Tokyo, Japan).

Immunofluorescent staining assay using the anti-FLAG monoclonal antibody (Sigma) was performed to detect the FLAG-tagged proteins as below. Following the fixation of the transfected cells with 2% paraformaldehyde at 25°C for 5 mins, the anti-FLAG antibody (1/200 in 0.5% BSA and PBS) was used as the first antibody. After incubating at room temperature for 1 h, the cells were rinsed 3 times with 200 μl prewarmed PBS plus 0.5% BSA and subsequently incubated with anti-mouse antibody conjugated with AlexaFluor 488 (Invitrogen) (1/200 in 0.5% BSA and PBS) at room temperature for 1 h. The images were captured using a confocal microscope (Olympus).

To evaluate the cell viability, staining with propidium iodide (PI) [30] was used. In the case of co-labeling with the HaloTag ligands, staining with AF488 was performed first, then PI staining for 15 min at room temperature with a final concentration of 2.5μg/ml followed. The cells were rinsed two times with PBS and images were analyzed as described above. In the case of co-staining with anti-FLAG monoclonal antibodies, PI staining was performed first, followed by labeling with the anti-FLAG monoclonal antibody.

To mimic the effect of the conformational changes of gp120 after its binding to the CD4 receptor, soluble CD4 was added to the 293FT cells transfected with HIV-1 Env expression vectors. The soluble CD4 protein (final concentration: 0.1 μM) was kept in the medium since immediately after transfection.

### Syncytia formation assay

A syncytia formation assay was performed by transfecting the HIV-1 Env expression vectors (listed as For mammalian system in Table 1) into the 293CD4 cells. The cells were transfected when they were about 50% confluent. At 48 h after transfection, the images were captured with IN Cell analyzer 1000 (GE Healthcare, Uppsala, Sweden). The fusion activity of Halo-fused Env was evaluated by counting 300 nuclei in total after staining with 2 μM Hoechst and determining the percentage of nuclei included in syncytia.

### Immunoblot analysis

Bacterial cultures (1 ml) were harvested and resuspended in 50 μl SDS-PAGE loading buffer (2% SDS, 2 mM DTT, 10% glycerol, 50 mM Tris-HCl, pH6.8, 0.01% Bromo phenol blue). The mixture was kept for 10 mins at 95°C and subjected to centrifugation (20,000 g, 4°C) with MX-301 (Tomy, Japan) to remove the pellets. Whole cell lysates (2 μl) were resolved using a 5-20% gradient SDS polyacrylamide gel (DRC, Tokyo, Japan). The proteins were transferred to the PVDF membrane and probed with 15,000-fold diluted anti-GFP antibody (Santa Cruz Biotechnology, Santa Cruz, USA) for 1 h at room temperature. Anti-mouse antibody (GE healthcare), diluted by 5,000-fold, was used as the secondary antibody. The signal was developed by streptavidin-biotinylated horseradish peroxidase complex (GE healthcare) and the chemiluminescence reagents (Roche), and detected by LAS3000 (Fuji).

The transfected 293FT cells grown in 10-cm dishes as described above were collected and centrifugated (5,000 g, 4°C) with MX-301. The cell pellet was lysed with 250μl of RIPA lysis buffer [50 mM Tris-Cl (pH 7.4), 150 mM NaCl, 1% NP-40, 0.1% SDS] and then centrifuged (MLA-130 rotor, 100,000rpm, 30 mins, 4°C) with Beckman Optima™Max Ultracentrifuge. The supernatant (20 μl) was treated with the same method as described above. The protein bands on the PVDF membrane were developed as described above, except for the 500-fold diluted anti-Halo pAb (Promega) and 5000-fold diluted anti-rabbit antibody (GE healthcare) which were used as the primary and secondary antibodies, respectively.

## Results

### Topology mapping of gp41 using GFP as a reporter in a prokaryotic system

We first employed the well-established prokaryotic topological analysis using GFP as a reporter [31,32]. If GFP is located in the cytoplasm it folds into an active form, whereas when it is translocated into the periplasm it is non-functional [31]. The periplasm-targeted maltose-binding protein was placed at the N-terminus of the gp41 portion to be tested, and then GFP, a topological reporter, was fused to the C-terminus of the gp41 fragment (Figure 2A). The series of gp41 proteins truncated at the different C-terminal positions were tested (Figure 1A and Table 1). The N-terminus of gp41 portion included was fixed at the position of 636th amino acid close to the predicted MSD (Figure 1A dotted line), because there is little controversy on the beginning of the MSD itself.

After transformation of *E. coli*. with one of the plasmids, the expression of the recombinant protein was evaluated by immunoblotting using an anti-GFP antibody and the results are shown in Figure 2B. The levels of protein expression with mpKMalp2e-gp41-1-GFP, mpKMalp2e-gp41-2-GFP, and mpKMalp2e-gp41-3-GFP, were low (Figure 2B), and we did not analyze these constructs further. The rest of constructs each expressed a comparable amount of the fusion protein of about 100kD (Figure 2B). The fluorescence intensities of GFP at 530 nm of *E. coli *induced for the expression of the fusion proteins were measured by a flow cytometry. Compared with the negative control that expresses GFP in the periplasm (mpKmalp2e-GFP), the GFP intensity adjusted by the cell density was significantly higher for mpKMalp2e-gp41-4-GFP, mpKMalp2e-gp41-5-GFP, mpKMalp2e-gp41-6-GFP mpKMalp2e-gp41-7-GFP and mpKMalp2e-gp41-8-GFP (Table 2). This suggested that GFP attached at the position 4 to 8 lies in the cytoplasm. Interestingly, there was no significant difference in the GFP fluorescent intensity adjusted by the level of the expression for mpKMalp2e-gp41-4-GFP, mpKMalp2e-gp41-5-GFP, mpKMalp2e-gp41-6-GFP, mpKMalp2e-gp41-7-GFP and mpKMalp2e-gp41-8-GFP. These data suggested that there was no topological shift of GFP reporter in these regions; therefore the Kennedy sequence and LLP regions are not exposed to the periplasmic region. These results are consistent with the single MSD model of gp41 (Figure 1B).

**Table 2:** Results of GFP quantification

Vector	Adjusted GFP signal (The number of counts /OD_600_)
optGFP_1-11_/pET-47md	4026.238

mpKMalp2e-gp41-4-GFP	1103.775

mpKMalp2e-gp41-5-GFP	971.453

mpKMalp2e-gp41-6-GFP	828.177

mpKMalp2e-gp41-7-GFP	1018.790

mpKMalp2e-gp41-8-GFP	986.997

mpKmalp2e-GFP	313.958

### Expression of HaloTag-attached HIV-1 Env in mammalian cells

Although the bacterial system is quick and informative, eukaryote specific post-translational modifications and/or the difference in the composition of lipids in the membrane may affect the topology of gp41. Therefore, HIV-1 Env with the C-terminus of gp41 linked to HaloTag was expressed in mammalian cells (Figure 3A). The HaloTag is a 33 kDa protein designed to covalently bind to its membrane-permeable/impermeable ligands conjugated with a fluorescent chromophore [33]. Based on the previous published results [11] and our own results of the prokaryotic system (see above), we focused on the analysis of the region after the predicted MSD of the single MSD model (truncation positions 4-8 in Figure 1A). The GPI-anchored HaloTag protein (Halo-GPI) and the HaloTag attached to the C-terminus of the Tac antigen after MSD (Tac-Halo) were made as the controls for the extracellular and intracellular positioning of HaloTags, respectively (Figure 3A and Table 1). Expression of HaloTag-attached envelope proteins was confirmed by immunofluorescence analysis with anti-gp120 antibody (data not shown) and immunoblotting analysis with anti-Halo antibodies (Figure 3B). The bands around 130-170kD and 40-55kD for pHIVenv-gp41-Halo are HaloTag-attached gp160 and gp41, respectively.

The membrane fusion capacity of these mutants was examined with a syncytia formation assay by transfecting the expression vector into 293CD4 cells [23]. Although the efficiency of the fusion was reduced in all of the HaloTag-attached envelope proteins, all still retained membrane fusion activity (Figure 3C). When we analyzed the fusion activity with the DSP assay [34], better fusion was observed (data not shown). Since the DSP assay relies on the smaller reporter proteins, the presence of the defect of pore dilatation in HaloTag attached mutants was suggested.

### Topology mapping of gp41 in mammalian cells using HaloTag-specific membrane-impermeable ligands

**Figure 4:**
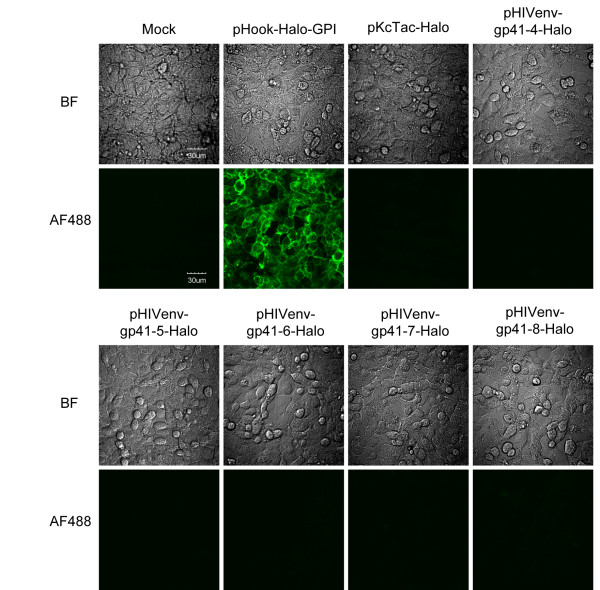
**Topological analysis of gp41 in 293FT cells**. Images of the cells stained with the membrane impermeable ligand, Alexa Fluor 488 (AF488), for HaloTag. The staining and image capturing were done at 44 h post transfection. Mock, mock DNA transfection. The names of expression vectors are shown. The bar indicates 30 μm.

The membrane-permeable and membrane-impermeable ligands with fluorescent chromophore available for HaloTag were used to examine the location of the attached HaloTag in relation to the cell membrane. Oregon Green (OG) that readily cross the cell membrane labels HaloTag located in both extracellular and intracellular spaces, whereas Alexa Fluor 488 (AF488), a membrane-impermeable ligand, should label HaloTag exposed on the cell surface. When we used the membrane-permeable substrate, OG, all of the 293FT cells transfected with HaloTag-fused truncated Env plasmids (pHIVenv-gp41-4-Halo, pHIVenv-gp41-5-Halo, pHIVenv-gp41-6-Halo, pHIVenv-gp41-7-Halo, and pHIVenv-gp41-8-Halo) were stained by the ligand (Additional File 1; Figure S1). The 293FT cells transfected with pHook-Halo-GPI and pKcTac-Halo were also stained by OG; the fluorescent signal was localized at the rim of the cells (Additional File 1; Figure S1). On the other hand, when we used the membrane-impermeable substrate, AF488, none of the 293FT cells transfected with the plasmids harboring HaloTag-fused Env with C-terminal truncation were stained (Figure 4 pHIVenv-gp41-4 to -8-Halo). As expected, the 293FT cells transfected with pHook-Halo-GPI were stained by AF488, but the 293FT cells transfected with pKcTac-Halo did not show any fluorescent signal under the same labeling and imaging conditions (Figure 4), verifying the authenticity of this experimental system. These results indicate that the HaloTag attached at positions 4 to 8 of gp41 are located in the cytoplasm of the cells. This result is consistent with the prokaryotic data (Table 2) and suggests that Kennedy sequence and LLP regions are both located in the cytoplasm, supporting the single MSD model of gp41 [11].

### Examination of membrane topology with HaloTag in syncytia formed in 293CD4

**Figure 5:**
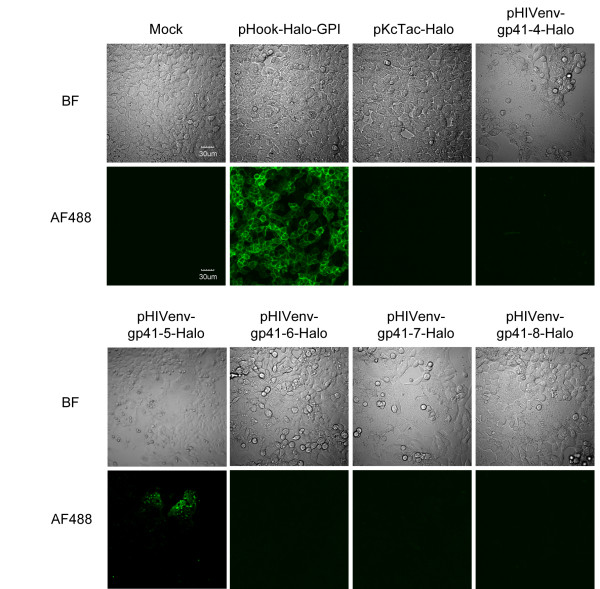
**Topological analysis of gp41 in 293CD4 cells**. Images of the cells stained with membrane impermeable ligand, Alexa Fluor 488 (AF488). The staining and image capturing were done at 20 h post transfection. The abbreviations used are same as in Fig. 4. The bar indicates 30 μm.

As the possibility for a transient topological change of gp41 during membrane fusion has been proposed [16,17], we induced the formation of syncytia in 293CD4 by transfecting a series of Env-HaloTag expression vectors and performed the labeling. All of the syncytia formed after transfecting the expression vector for each Env-HaloTag were positively stained with OG, membrane-permeable ligand, during membrane fusion, confirming the expression of Halo-fused Envs (Additional File 2; Figure S2). When the membrane-impermeable ligand AF488 was used for staining, most of the multinucleated 293CD4 cells expressing various gp41 truncation mutants were not stained (Figure 5). The only exception was the cells transfected with pHIVenv-gp41-5-Halo, in which rare and weak staining of the syncytia were observed (Figure 5). Even the later time points with the similar levels of syncytia formation with pHIVenv-gp41-8-Halo were chosen to compensate the reduced fusion efficiency of pHIVenv-gp41-5-Halo, the staining incidence for pHIVenv-gp41-5-Halo did not increase. The 293CD4 cells transfected with the control plasmids, pHook-Halo-GPI (HaloTag on the cell surface) and pKcTac-Halo (HaloTag in the cytoplasm), showed the results consistent with their expected topological locations (Figure 5 pHook-Halo-GPI and pKcTac-Halo).

When the 293CD4 cells transfected with pHIVenv-gp41-5-Halo were stained with the anti-HaloTag antibody without permeabilization procedure, rare events of staining were observed (data not shown). These results suggest that the possibility of sporadic exposure of cytoplasmic domain of gp41 during membrane fusion with pHIVenv-gp41-5-Halo.

### Augmented membrane permeability by Env-induced membrane fusion

The result shown above for pHIVenv-gp41-5-Halo could be an indication of a rare translocation of the cytoplasmic region of the gp41. The reason why the translocation, if happening, is limited to the truncation at position 5 with a very low incidence was not clear. Since there was no staining for pHIVenv-gp41-4-Halo, we have to assume a hypothetical MSD between the position 4 and 5. This is to assume the Kennedy region to be the hypothetical MSD and is different from the model shown in Figure 1C. The hydrophilic Kennedy sequence is not likely to be an MSD by several prediction algorithms (Table 3). An alternative possibility is that the sporadic staining was due to the induced permeability of membranes in syncytia.

**Table 3:** Computational analyses of possible transmembrane domain

Program	Region of the predicted membrane-spanning segment (original: 684-706)
TroPred	684-705

TMHMM	678-701

SOSUI_MP1	675-708

SOSUI	683-706

**Figure 6:**
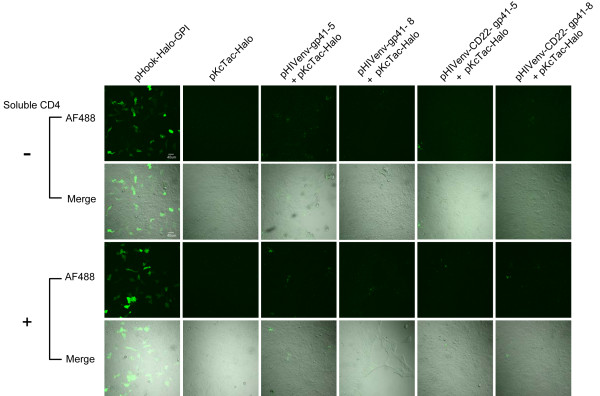
**Staining of 293FT cells cotransfected with the Env expression vector and pKcTac-Halo in the presence or absence of soluble CD4 by membrane-impermeable ligand, Alexa Fluor 488 (AF488)**. Soluble CD4 (0.1 μM) was used to induce the conformational changes of gp120. The names of the expression vectors used were shown on top. Merge: merged images of bright field and AF488 signals. The bar indicates 40 μm.

To distinguish the alteration of gp41 topology from membrane permeability induced during membrane fusion, we co-expressed tag-free HIV-1 Env together with Tac-Halo in the same cells. Namely, the pKcTac-Halo, and pHIVenv-gp41-5/pHIVenv-gp41-8 or pHIVenv-CD22-gp41-5/pHIVenv-CD22-gp41-8 (Table 1) were co-transfected simultaneously. We then probed the HaloTag expressed in the cytoplasmic side (see Figure 3A) with AF488, membrane-impermeable ligands. Both 293FT (fusion incompetent) and 293CD4 (fusion competent) cells were used to determine the effect of membrane fusion. The co-transfected 293FT cells were not stained with AF488 (Figure 6 -soluble CD4), whereas these cells were stained with OG (data not shown). The expressions of Env in 293FT cells were confirmed by immunoblotting (Additional file 3; Figure S3). The addition of soluble CD4, which can induce the early conformational change of gp120, did not show any changes in the staining patterns (Figure 6 + soluble CD4).

**Figure 7:**
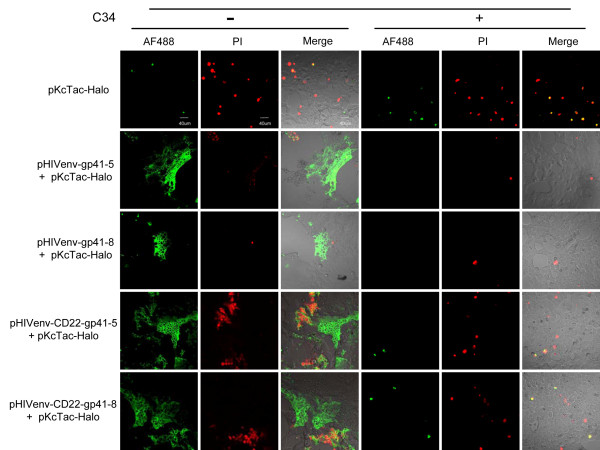
**Examination of the membrane permeability during membrane fusion**. Staining of 293CD4 cells cotransfected with the Env expression vector and pKcTac-Halo with membrane-impermeable ligand, Alexa Fluor 488 (AF488, shown in green) in the presence (+) or absence (-) of the fusion inhibitor, C34 (2 μM). The names of the expression vectors used are shown in left. Staining and image capturing were performed as described in Methods. For the HIV-1 Env with its native MSD images were captured at 16 h post transfection. The similar assay was performed for CD22MSD mutants at 44 h post transfection. PI: images with propidium iodide staining as described in Methods. Merge: merged images of bright field with AF488 and PI labelings. The bar indicates 40 μm.

In the case of 293CD4 cells, however, the co-transfected cells (Figure 7 -C34, pHIVenv-gp41-5 or 8 + pKcTac-Halo,) could be clearly stained by AF488 at the site of syncytium (Figure 7 -C34). These staining were not due to the cell death, because some cells labeled with AF488 did not show the staining with propidium iodide (Figure 7 shown in red). The staining with AF488 was abolished when membrane fusion was inhibited by the addition of C34, an inhibitor of six-helix bundle formation (Figure 7 +C34, pHIVenv-gp41-5 or -8 +pKcTac-Halo). These results indicated that the induction of the permeability was dependent on active membrane fusion.

**Figure 8:**
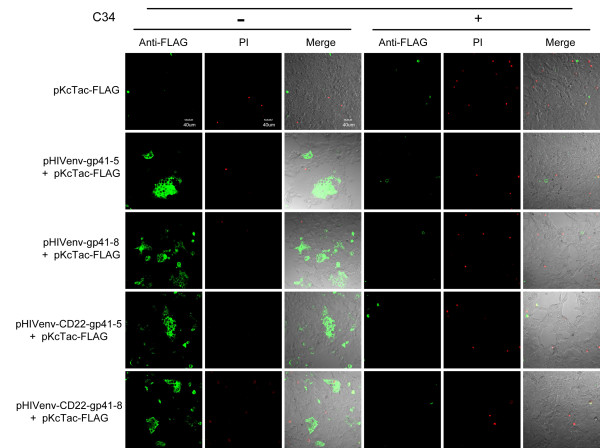
**Examination of membrane permeability during membrane fusion using antibodies as probes**. Staining of 293CD4 cells cotransfected with the Env expression vector and pKcTac-FLAG in the presence (+) or absence (-) of C34 by anti-FLAG antibody. The pKcTac-FLAG vector expresses the Tac proteins tagged with FLAG at their C-termini. Anti-FLAG: images after staining with the anti-FLAG antibody as described in Methods are shown in green. The time points for the assay were same as Fig. 7 (16 h post transfection for wild type MSDs and 44 h for CD22MSDs, respectively). PI: Images after propidium iodide staining. Merge: merged images of Anti-FLAG and PI stainings together with that of bright fields. The concentration of C34 was 2 μM. The names of the expression vectors used are shown in left. The bar indicates 40 μm.

To examine whether the observed membrane permeability during membrane fusion allows antibodies to penetrate membranes, we probed the 3 × FLAG epitope attached to the cytoplasmic portion of the Tac antigen (Tac-FLAG) with the anti-FLAG antibody. The intracellular 3 × FLAG tag was detectable when HIV-1 Env with or without the truncation, pHIVenv-gp41-5 and pHIVenv-gp41-8, respectively, were co-expressed (Figure 8 -C34). Although the staining pattern of each syncytium varies, it seemed that the incidence of the positively stained syncytia was slightly lower than that obtained with the membrane-impermeable ligands shown in Figure 7. When the membrane was permeabilized with detergent prior to antibody staining, all of syncytia were stained well (data not shown). These results suggest both the full-length and truncated Env have the ability to permeabilize the membrane to allow the antibodies to cross the membranes.

### Augmented membrane permeability is dependent on MSD sequence

Since membrane permeability was induced in the cells transfected with pHIVenv-gp41-5-Halo, the presence of LLPs is not required for the increased permeability. To further characterize the region required for this enhanced permeability we constructed the mutants in which the original gp41 MSD was replaced with the foreign MSD derived from CD22 [27] in the pHIVenv context. Previous reports indicated that the MSD derived from CD22 did not alter the function of HIV-1 Env [27]; however, the replacement seemed to delay the appearance of syncytia when compared with the wild type (see below). We compared these mutants with the HIV-1 Env with the native MSD. In the case of the HIV-1 Env with its native MSD, intracellular HaloTag was detectable with membrane-impermeable AF488 at the earlier time point after co-transfection (16 h post transfection, Figure 7; pHIVenv- gp41-5 and 8). On the other hand, there was minimal staining in cells co-transfected with HIV-1 Env with CD22 MSD at 16 h after transfection (data not shown). At 44 h post transfection when the cells transfected with the native gp41 MSD were almost gone due to the cell death, some cells transfected with CD22 MSD mutants were stainable with AF488 (Figure 7 -C34, pHIVenv-CD22-gp41-5 or 8 + pKcTac-Halo). Therefore there is a significant difference in the pattern of the staining between the native and CD22 MSDs. At 44 h post transfection, there were more dead cells as indicated by the positive PI staining. These cells were also stained with AF488. There are, however, some syncytia stained only with AF488 for CD22 MSD mutants (Figure 7). Inhibition of the membrane fusion with C34 blocked the staining (Figure 7 +C34). Similar results were obtained if anti-FLAG antibodies were used to detect the FLAG tag located in the cytoplasm. (Figure 8 pHIVenv-CD22-gp41-5 or 8 + pKcTac-FLAG). Taken together, these results indicated that the induction of permeability was membrane fusion-dependent and that the gp41 MSD played some role in the degree of induced permeabilization during membrane fusion.

## Discussion

In this study we examined the membrane topology of the gp41 subunit in two different biological systems. The truncated gp41 subunit was tagged with the topological reporter protein at the C-terminus (Figure 1, 2, 3). A prokaryotic reporter, GFP [31,32] and mammalian reporter, HaloTag [33], were used. Both reporters enabled us to examine the topology in living cells without the artifacts caused by fixing.

In our prokaryotic system, all of the tested constructs (mpKMalp2e-gp41-4, 5, 6, 7- and 8-GFP) showed stronger GFP fluorescence than the control. This suggested that gp41 had a single MSD that places the Kennedy sequence, LLP-2, LLP-3, LLP-1 and the C-terminus of gp41 in the cytoplasmic side. The analysis with β-lactamase, another topology reporter, which is only active in periplasm produced the data consistent with that of GFP (data not shown). These data are consistent with the results obtained by the currently available several programs for prediction of transmembrane domains (Table 3).

Our analysis of gp41 topology in mammalian cells without membrane fusion (293FT cells) supported the single transmembrane model, concordant with that of the prokaryotic system. Only sporadic staining with membrane impermeable ligands was observed for pHIVenv-gp41-5-Halo (truncation at position 747 in HXB2 Env) in fusion-competent 293CD4 cells. Since staining for the preceding truncation point, pHIVenv-gp41-4-Halo (truncation at the residue 725 in HXB2), was negative, suggesting an additional MSD between the residue 725 and 747. This region contains about 20 mainly hydrophilic amino acid residues that correspond to the Kennedy sequence. As shown in Table 3 no MSD was predicted in this region by several computational algorithms. So we speculated that there is other reason for the observed apparently contradictory observations.

We examined the possibility of enhanced membrane permeability to account for the staining of putative intracellular regions during membrane fusion. The intracellularly located HaloTag was labeled using the membrane-impermeable ligand for HaloTag when HIV-1 Env-mediated membrane fusion occurred (Figure 7). Antibodies were also able to stain the intracellular targets in conditions of membrane fusion (Figure 8). Although we cannot completely exclude the possible alternations of the gp41 topology, the multiple MSD model based on the epitope mapping [16,17] needs to be reevaluated carefully given the increased permeability we have observed.

We attempted to map the region(s) of gp41 responsible for increased membrane permeability. Our data suggested that the increased permeability was dependent on active membrane fusion (Figure 7 and 8). It is consistent with several reports of the fusion-dependent induction of membrane permeability in HIV-1 infected cells [35-38]. It is known that the several isolated subdomains of gp41, critical for membrane fusion, have potential to permeabilize the mammalian and the bacterial membranes [39-50]. These include the fusion peptide, the membrane proximate external region, the MSD and the LLPs. Our data of pHIVenv-gp41-5 (Figure 7 and 8) excludes the possibility of the role of LLPs. Our data using MSD replacement mutants pHIVenv-CD22-gp41-5 or pHIVenv-CD22-gp41-8, suggested the MSD affected the level of permeability, but whether the MSD directly affected the permeability or the effect was mediated via the efficiency of the membrane fusion was hard to be determined.

Since our assay relied on the topological reporter proteins attached at the C-termini of truncated gp41 proteins, the possibility of artifacts cannot be excluded. The exact reason why neutralizing epitopes mapped to the cytoplasmic regions remains unclear. There are at least two possible explanations. First, it is possible that the some of the antibodies themselves are intrinsically membrane permeable. Second, permeability that is sufficient to permit antibodies to cross the membrane may be induced by membrane fusion. Although our findings support the latter model, further study is needed to explore whether an alternative topology for gp41 MSD during membrane fusion really takes place and if such a possibility is a general phenomenon for other HIV-1 strains.

## Conclusions

The membrane topology of the gp41 subunit of HIV-1 Env was examined in both prokaryotic and mammalian systems. The topology with a single MSD was supported in both systems. In addition, augmented membrane permeability was shown to be dependent on both the sequence of MSD and active membrane fusion.

## List of abbreviations

Env: envelope glycoprotein; HIV-1: human immunodeficiency virus type-1; LLP: lentiviral lytic peptide; MSD: membrane-spanning domain; GFP: green fluorescent protein; GPI: glycosylphosphatidylinositol; FBS: fetal bovine serum; OG: Oregon Green; AF488: Alexa Fluor 488; PI: propidium iodide; PBS: phosphate buffered saline.

## Competing interests

The authors declare that they have no competing interests.

## Authors' contributions

SL, NK, YL and DX performed the experiments. The analysis in the prokaryotic system was done by SL and DX. The work in mammalian system was performed by SL, NK and YL. The study was conceived by ZM. AI supervised the entire work. SL, NK and ZM wrote the manuscript. All authors read and approved the final manuscript.

## Acknowledgements

This work was supported by a contract research fund from the Ministry of Education, Culture, Sports, Science and Technology for Program of Japan Initiative for Global Research Network on Infectious Diseases. We thank Dr. Kunito Yoshiike for his critical reading of the manuscript.

## Supplementary Material

Additional file 1 **Supplemental Fig.1 Detection of HaloTag-attached HIV-1 Env in 293FT cells**. Images of the transfected 293FT cells stained with membrane-permeable ligand, Oregon Green (OG). BF indicates the bright filed images. The names of the expression vectors are shown. Mock, mock DNA transfection. Click here for file

Additional file 2 **Supplemental Fig.2 Detection of HaloTag-attached HIV-1 Env in 293CD4 cells**. Images of the transfected 293CD4 cells stained with membrane permeable ligand, Oregon Green (OG). The nomenclature used are same as Supple Figure 1. Click here for file

Additional file 3 **Supplemental Fig.3 Expression of HIV-1 Env with its native MSD or foreign CD22MSD in 293FT cells**. The expression of the envelope protein was examined by immunoblotting using the anti-gp120 antibody as described previously [23]. The names of the expression vectors were shown on top. The bands of gp160 and gp120 are indicated. Click here for file
