# Supplementary figures and images for: Membrane topology analysis of HIV-1 envelope glycoprotein gp41
Source: Retrovirology. 2010 Nov 30;7:100. doi: 10.1186/1742-4690-7-100 (PMC3012657; doi:10.1186/1742-4690-7-100)

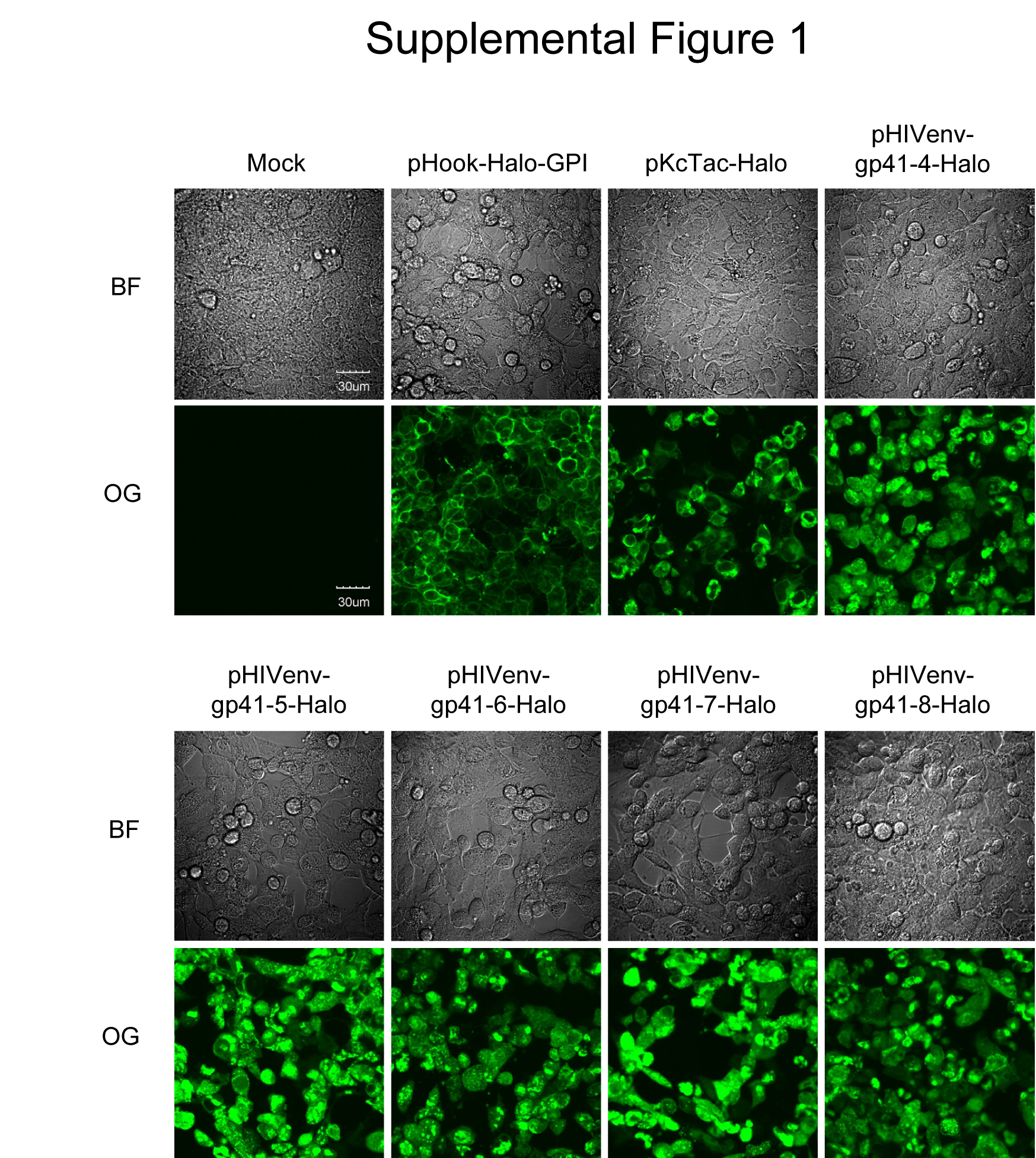

Supplement: Additional file 1 — Supplemental Fig.1 Detection of HaloTag-attached HIV-1 Env in 293FT cells. Images of the transfected 293FT cells stained with membrane-permeable ligand, Oregon Green (OG). BF indicates the bright filed images. The names of the expression vectors are shown. Mock, mock DNA transfection. [file 1742-4690-7-100-S1.TIFF]

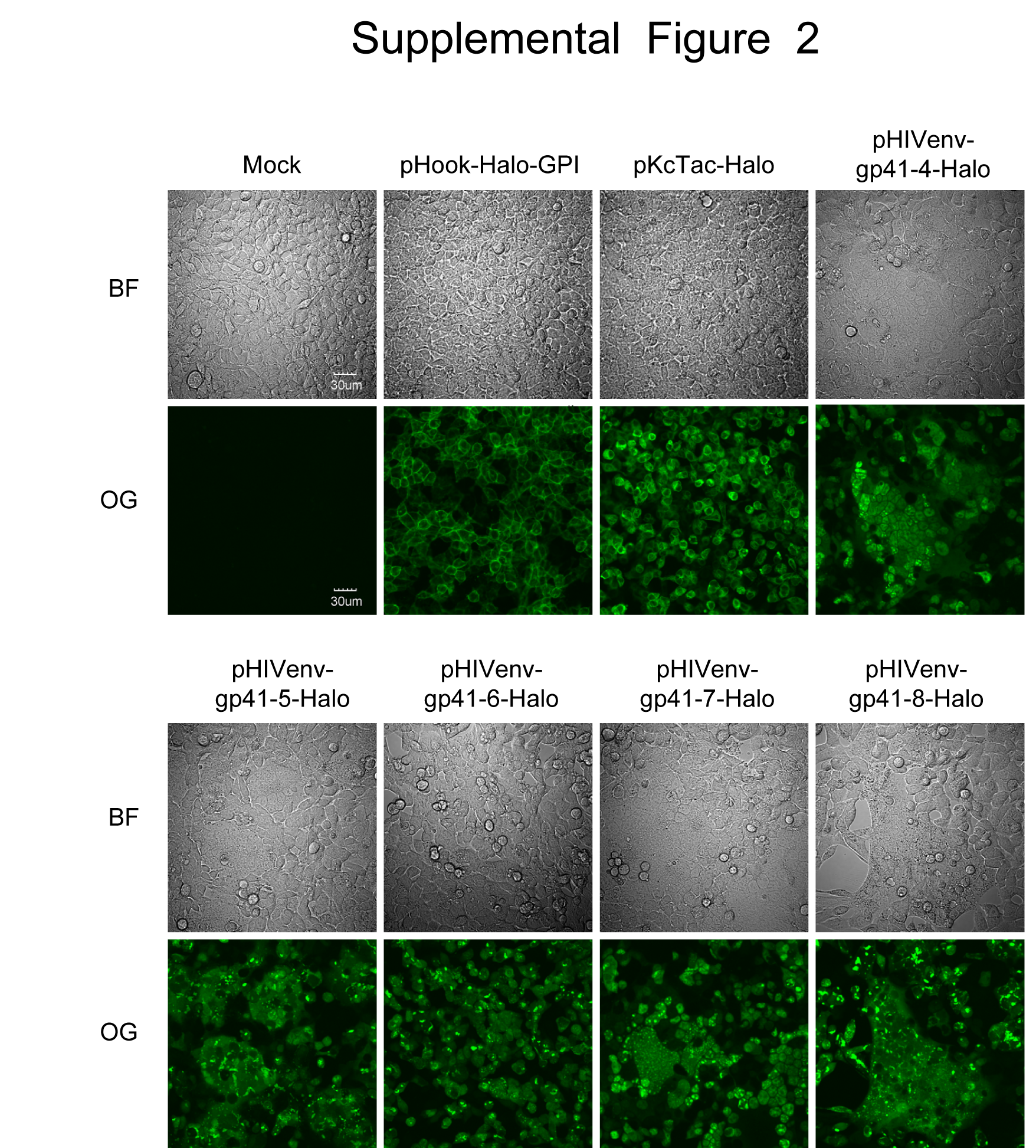

Supplement: Additional file 2 — Supplemental Fig.2 Detection of HaloTag-attached HIV-1 Env in 293CD4 cells. Images of the transfected 293CD4 cells stained with membrane permeable ligand, Oregon Green (OG). The nomenclature used are same as Supple Figure 1. [file 1742-4690-7-100-S2.TIFF]

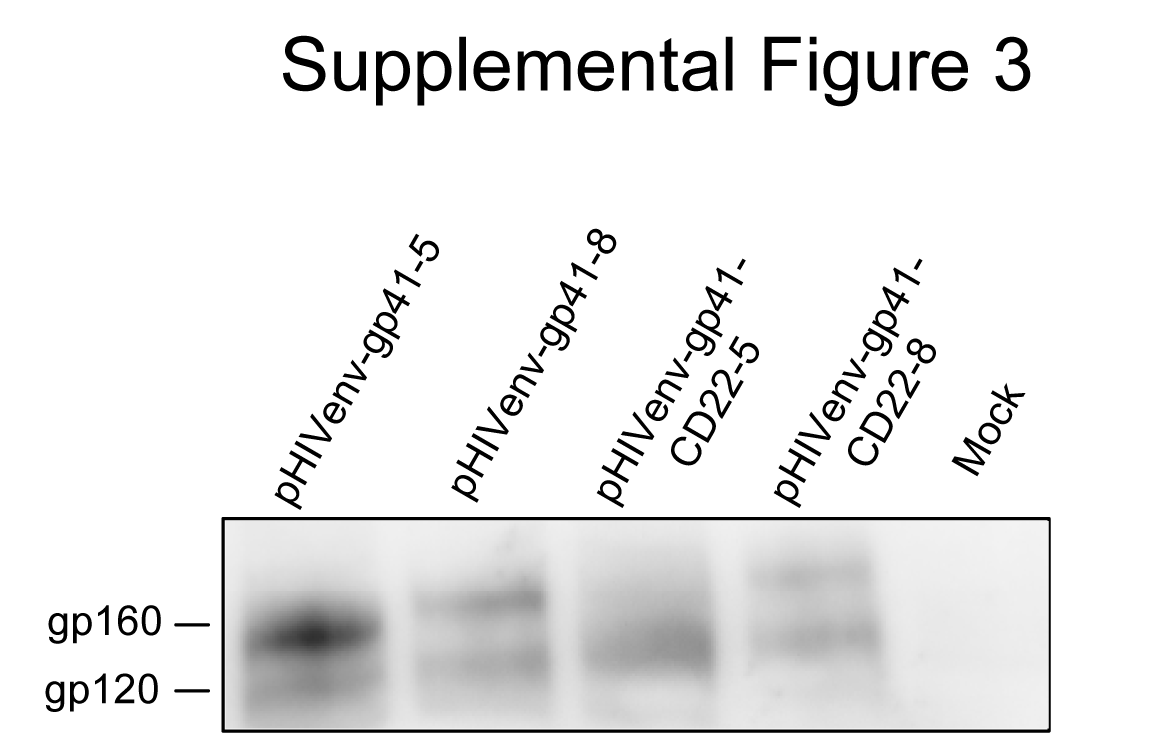

Supplement: Additional file 3 — Supplemental Fig.3 Expression of HIV-1 Env with its native MSD or foreign CD22MSD in 293FT cells. The expression of the envelope protein was examined by immunoblotting using the anti-gp120 antibody as described previously [23]. The names of the expression vectors were shown on top. The bands of gp160 and gp120 are indicated. [file 1742-4690-7-100-S3.TIFF]
